# In Situ Spectroscopic and Electrical Investigations of Ladder-type Conjugated Polymers Doped with Alkali Metals

**DOI:** 10.1021/acs.macromol.2c01190

**Published:** 2022-08-15

**Authors:** Yongzhen Chen, Han-Yan Wu, Chi-Yuan Yang, Nagesh B. Kolhe, Samson A. Jenekhe, Xianjie Liu, Slawomir Braun, Simone Fabiano, Mats Fahlman

**Affiliations:** † Laboratory of Organic Electronics, Department of Science and Technology, Linköping University, Norrköping 60174, Sweden; ‡ Department of Chemical Engineering and Department of Chemistry, University of Washington, Seattle, Washington 98195-1750, United States

## Abstract

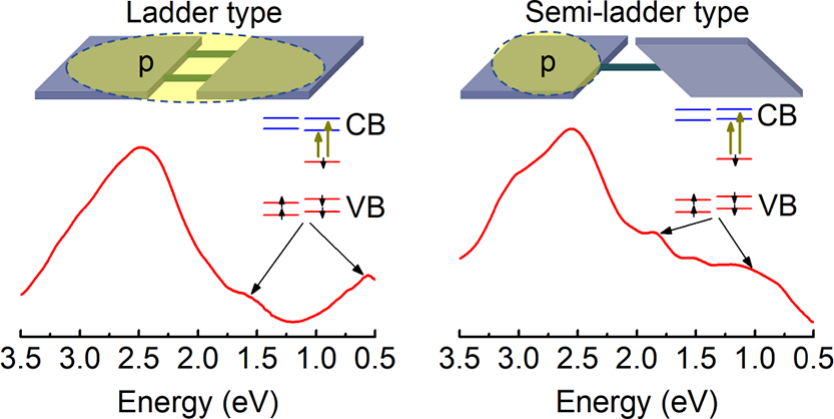

Ladder-type conjugated polymers exhibit a remarkable performance in (opto)electronic devices. Their double-stranded planar structure promotes an extended π-conjugation compared to inter-ring-twisted analogues, providing an excellent basis for exploring the effects of charge localization on polaron formation. Here, we investigated alkali-metal n-doping of the ladder-type conjugated polymer (polybenzimidazobenzophenanthroline) (BBL) through detailed in situ spectroscopic and electrical characterizations. Photoelectron spectroscopy and ultraviolet–visible–near-infrared (UV–vis–NIR) spectroscopy indicate polaron formation upon potassium (K) doping, which agrees well with theoretical predictions. The semiladder BBB displays a similar evolution in the valence band with the appearance of two new features below the Fermi level upon K-doping. Compared to BBL, distinct differences appear in the UV–vis–NIR spectra due to more localized polaronic states in BBB. The high conductivity (2 S cm^–1^) and low activation energy (44 meV) measured for K-doped BBL suggest disorder-free polaron transport. An even higher conductivity (37 S cm^–1^) is obtained by changing the dopant from K to lithium (Li). We attribute the enhanced conductivity to a decreased perturbation of the polymer nanostructure induced by the smaller Li ions. These results highlight the importance of polymer chain planarity and dopant size for the polaronic state in conjugated polymers.

## Introduction

Conducting polymers are widely investigated in organic (opto)electronic devices for energy conversion and storage.^1,2^ Their unique advantages of versatility, solution processability, and flexibility successfully meet the demands of large-scale and wearable devices.^3^ The charged states, that is, polarons or bipolarons, formed by the gain/loss of electrons on the conjugated chains play a central role in the operation of such devices and directly influence the electrical conductivity, optical absorption, and thermoelectric properties of the polymer films.^4−7^ Therefore, understanding the electronic structure of polarons/bipolarons in doped conjugated polymers could help in the design of novel materials and boost device performance.

Many experimental and theoretical studies have been conducted on conjugated polymers and their corresponding oligomers, yielding rich information about the nature of the charged conjugated chains.^8−11^ However, these studies are primarily based on the classical conjugated polymers with a single-stranded linkage between repeat units, such as polythiophene, polyfluorene, poly(phenylene vinylene), and their derivatives. In comparison, ladder-type conjugated polymers exhibit lower intrachain torsional disorder and have enhanced π–π stacking due to their double-stranded structure.^12,13^ Polarons on such rigid-chain conjugated polymers are attracting more and more attention due to the increasing application of the materials in organic electronics.^14−20^ Previously, the spectroscopic and electrical properties of chemically and electrochemically doped poly(benzimidazobenzophenanthroline) (BBL) have been extensively explored.^21−23^ Density functional theory (DFT) calculations have also been carried out to explain the experimental observations by modeling the multiple charged states of the oligomer.^24−26^ However, additional studies are needed to achieve a thorough understanding of the doping-induced properties. One severe limitation is the sparse experimental data on chemically doped BBL. There is a lack of reports on (i) the evolution of the low-energy band in the UV–vis–NIR spectrum upon different doping levels, especially at high doping levels; (ii) the photoelectron spectroscopy (PES) tracking of the changes in the valence band and core levels; and (iii) the influence of different dopants on the polaron formation and resulting optoelectronic properties.

It is difficult to detect the UV–vis–NIR at high doping levels and the polaronic state in ultraviolet photoelectron spectroscopy (UPS) upon chemical doping. An important reason is that the doping efficiency of previously used small-molecule and polymeric dopants (e.g., TDAE, *N*-DMBI, PEI, etc.) is not high enough, or few of the charge carrier pairs dissociate into free polarons after charge transfer.^27−31^ The low concentration of polarons makes the new states hard to resolve from the highest occupied molecular orbitals of the host polymers and dopants in the UPS spectrum. Using alkali metals as the n-type dopant is an effective method to improve the doping efficiency as their low ionization potential enables efficient charge transfer to the polymers.^32^ The interactions between alkali metals and polymers used as the active light-emitting layer have been widely studied, where polaron and/or bipolaron states are observed near the Fermi level.^33−35^ Due to the high sensitivity of the alkali metals to air, however, the doped film requires ultrahigh vacuum (UHV). Thus, it is difficult to perform other characterizations after the UPS measurement.

**Figure 1:**
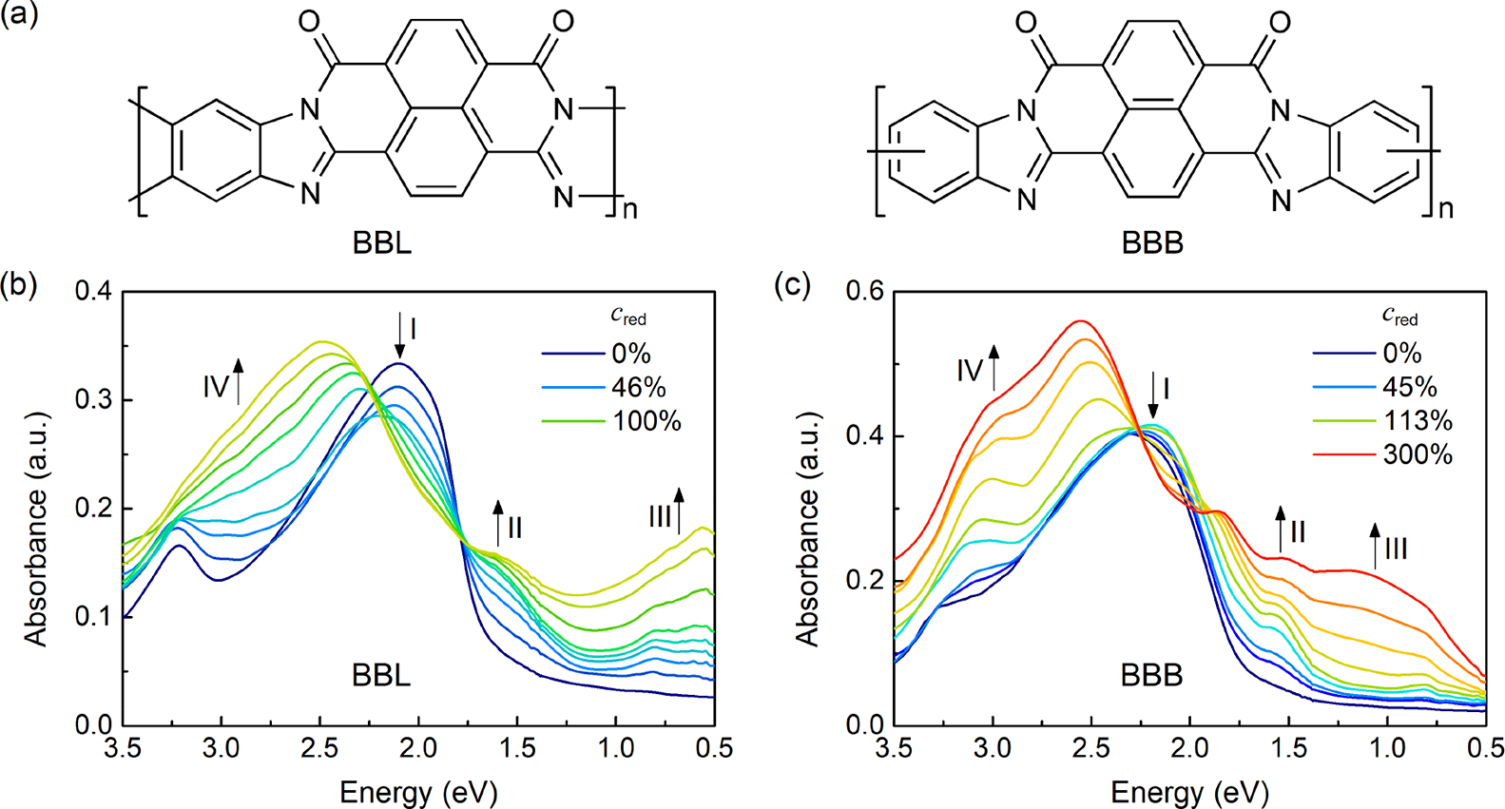
(a) Molecular structures of BBL and BBB. UV–vis–NIR spectra of potassium-doped (b) BBL and (c) BBB films with an incremental increase in the doping ratio. The doping ratio *c*_red_ refers to the number of K atoms per repeat monomer.

In the present work, we integrate the optical and electrical measurements with PES to collect all information without breaking the UHV. As we reported recently,^36^ the valence structure from UPS measurement directly defines the occupied polaron states, and the UV–vis–NIR absorption spectrum provides the optical transition energies and indirect data on the unoccupied states. The combined UPS and UV–vis–NIR spectra enable the formation of a complete energy level diagram. In combination with the reported DFT simulations, we systematically investigate the polaronic structure of BBL at different doping levels by progressively increasing the amount of alkali metals. To clarify the influence of the rigid-chain ladder structure of BBL, we performed a complementary study on the semiladder analogue BBB with a single-stranded structure (see Figure 1a), showing a more localized nature of the polaronic states in BBB. In addition, we explored the different interactions between the conjugated polymers and dopants by replacing K (potassium) with Li (lithium), which shows a more covalent interaction when BBL is doped by Li rather than K.

## Experiments

### Film Preparation and Doping

BBL and BBB films used in this work were made from ethanol dispersions using the spray-coating method. The thickness of all films is maintained at 40–50 nm. The substrate used for the PES measurements was indium tin oxide-coated glass, while for the optical and electrical measurements, we used bare glass. All substrates were cleaned with detergent and ultrasonicated sequentially in water, acetone, and isopropanol before use. The BBL and BBB samples had intrinsic viscosity of 11.6 dl g^–1^ (*M*_w_ = 60.5 kDa) and 1.38 dl g^–1^ (*M*_w_ = 59.5 kDa) in methane sulfonic acid at 30 °C, respectively.

The in situ doping process was carried out by evaporating potassium or lithium from a SAES (SAES getters S.p.A, Italy) getter source in the UHV preparation chamber (ca. 5 × 10^–9^ mbar), as shown in Figure S1. After exposure to the alkali vapor, the samples with different substrates were transferred to the analysis chamber and optical cavity. The doping ratio, *c*_red_ (here defined as the number of potassium or lithium atoms per monomer, e.g., *c*_red_ = 100% means one K/Li atom per monomer unit), was deduced from the X-ray photoelectron spectroscopy (XPS) result by integrating the peak areas of K 2p/Li 1s and C 1s and applying appropriate atomic sensitivity factors.

### In Situ Spectroscopic and Electrical Characterizations

UPS was performed in a UHV surface analysis system equipped with a Scienta-200 hemispherical analyzer. The excitation source for UPS was a standard He-discharge lamp with *h*v = 21.22 eV (He I), and for XPS, monochromatized Al Kα radiation with 1486.6 eV energy was used. The UV–vis–NIR absorption spectra were measured in an optical cavity connected to the UHV system (see Figure S1) and using the spectrometers Ocean Optics FLAME-T-VIS-NIR-ES (350–1000 nm) and NIR QUEST512–2.5 (900–2500 nm). The full–spectrum curve was a combination of two parts joined at 900 nm. The electrical conductivity measurement was performed inside the UHV chamber using a Keithley 2636B Source Meter. The electrodes were fabricated by depositing a 5 nm titanium (Ti) adhesion layer and 50 nm Au on glass before active layer deposition, which had a channel length/width (*L*/*W*) of 30 μm/1000 μm.

## Results and Discussion

### UV–vis–NIR Characterization

First, we investigated the evolution of UV–vis–NIR absorption spectra of BBL and BBB at different doping levels by changing the K vapor exposure time. The measurements were carried out between the stepwise deposition of K. The doping ratio *c*_red_, defined as the number of K atoms per monomer unit, is deduced from the XPS spectra by calculating the sensitivity-factor-adjusted K/C area ratio. As shown in Figure 1, the absorption spectra of both pristine BBL and BBB are characterized by two distinct bands, that is, a UV band above 3.0 eV and a visible band (labeled I). The latter is attributed to the π–π* transition and is centered at 2.1 eV for BBL and 2.3 eV for BBB, respectively, indicating a narrower optical band gap for BBL than BBB, which stems from a more planar backbone structure.^15^

Although the optical features of the neutral films are very similar, n-type doping causes a significant difference between the two materials. For BBL (Figure 1b), K doping is accompanied by the generation of two new absorption bands in the near-infrared region. One band is observed around 1.6 eV (labeled II), and the other starts at ca. 1.0 eV and extends into the mid-infrared region (labeled III). The intensity of these two bands increases simultaneously with increasing the K content. According to time-dependent density functional theory (TD-DFT) calculations, these two bands are mainly attributed to transitions from new gap states to unoccupied orbitals, with band II attributed to both *cis* and *trans* conformers and band III attributed only to the *trans* conformer, whose structures are shown in Figure S2.^25,26^

At *c*_red_ ≤ 50%, a new feature IV appears at approximately 3.0 eV, along with the bleaching of the neutral state absorption, in agreement with the previous observation for BBL doped with molecular or polymeric dopants.^19,20,23^ This change is ascribed to a replacement of the frontier orbitals in the charged polymers. More details about this feature can be observed from the absorption difference spectra. As shown in Figure S3a, a shoulder located at 2.85 eV is visible in addition to the main peak. Both peaks become slightly red-shifted during the sequential deposition of K. However, by further increasing the doping ratio (*c*_red_ > 50%), band I at ca. 2.1 eV, which arises from the neutral polymer, gradually blue-shifts, strengthening and reaching 2.5 eV at the final doping stage. This evolution strongly resembles the spectral variation produced by electrochemical doping at high reduction potentials^21^ but has never been observed in chemical doping. This is due to the superior reducing character of K compared to organic molecules and polymers. Similar results are also observed when replacing K with another strong dopant Li, as presented in Figure S4. Compared to K, band III in the Li-doped BBL film does not grow together with band II. Instead, it emerges at high doping ratios (∼200%), accompanied by a decrease in the optical feature at around 2.5 eV.

In the case of BBB, the evolution of the absorption spectrum upon K doping is totally different from BBL (see Figure 1c). First, the doping ratio required to produce a similar change in the spectrum of K-doped BBB films is much higher, as observed for the other characterizations reported below. Second, the NIR bands II and III are narrower than that in BBL. The intensity of the low-energy band III is small up to a doping ratio of 113%, similar to Li-doped BBL, and its maximum blue-shifts to 1.0 eV. The neutral band I (2.3 eV) shows a slight red-shift and broadening at low doping ratios (*c*_red_ < 100%), giving rise to the multi-peak structure in the range 1.6–2.25 eV (see the absorption difference spectra in Figure S3b). With the bleaching of the neutral band at high doping ratios, the multi-peak structure shrinks to a narrow peak located at 1.75 eV. In addition, feature IV near 3.0 eV is always shown as an intense peak rather than a structureless feature as in BBL. As a result, this feature in the absorption difference spectra becomes stronger and separates from the peak at around 2.5 eV. From the comparison, we know that although the analogous structure enables BBB to maintain a similar enhanced absorption in the blue and NIR regions upon K doping, the evolution of specific peaks varies greatly in intensity and energy. These differences are attributed to the distorted (twisted) backbone of BBB that interrupts polaron delocalization along the polymer chain.

### Ultraviolet Photoelectron Spectroscopy Characterization

**Figure 2:**
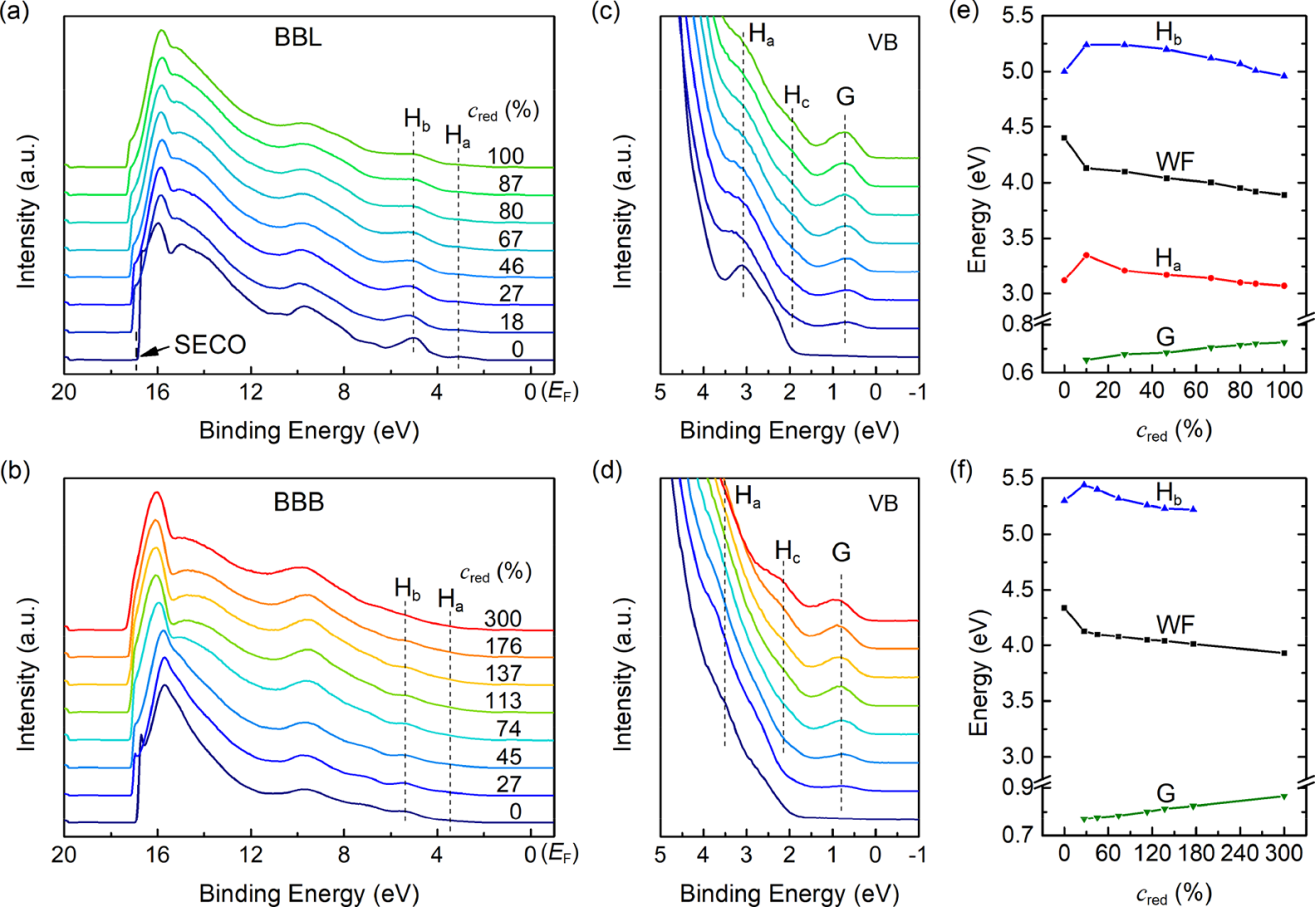
Evolution of the UPS spectra with increasing K doping ratio for (a) BBL and (b) BBB and the (c,d) corresponding enlarged spectra in the valence band region. All spectra are aligned to the Fermi level (*E*_F_). Work function (WF = *hv*–SECO), valence band (*H*_a_ and *H*_b_), and polaronic state (G) changing with the doping ratio are derived from the spectra for (e) BBL and (f) BBB. Due to the H_a_ peak in BBB overlaps *H*_b_, the data of *H*_a_ are not present here.

Next, we performed UPS measurements to explore the change in electronic structures in the doped polymers. Figure 2a,b presents the UPS spectra of BBL and BBB with incremental deposition of K. The work functions of the pristine films are quite similar, that is, 4.40 eV for BBL and 4.34 eV for BBB. We observe an obvious decrease in the work function for both polymers upon the first doping step, which keeps decreasing slowly at higher doping levels (see Figure 2e,f).

The valence band spectrum of the pristine BBL includes two clear and separate peaks below the Fermi level (*E*_F_), that is, *H*_a_ and *H*_b_, with maxima of 3.1 and 5.0 eV, respectively. According to DFT simulations, *H*_a_ consists of several orbitals located over the whole monomer unit with a π character, while *H*_b_ originates mainly from orbitals located only on the benzimidazole unit.^25^ After the first deposition of K (*c*_red_ = 18%), both peaks move away from the Fermi level by 0.25 eV with decreased intensity and sharpness, and a new peak (labeled G) appears at 0.66 eV below the *E*_F_, as depicted in the valence band spectra in Figure 2c. The new peak corresponds to the polaronic state filled with an electron transferred from K. By increasing the doping level, *H*_a_ and *H*_b_ peaks shift slowly toward *E*_F_, along with a remarkable enhancement of the polaronic feature. In addition, another new peak (labeled *H*_c_) at 1.9 eV is observed at higher doping ratios, which partially overlaps with the *H*_a_ peak. Both the new-formed features (G and *H*_c_) shift to higher binding energy upon a gradual increase in the K content (the maximum energy of G is shown in Figure 2e).

The valence band spectrum of pristine BBB shown in Figure 2d is similar to BBL. However, all peaks are broadened due to the semiflexible BBB backbone, disrupting the polymer chain arrangement and affecting the intra-chain π-conjugation lengths. As a result, peak *H*_a_ overlaps entirely with *H*_b_. Upon K doping, we observed a similar shift (see Figure 2f) and attenuation for the valence band features as for BBL, along with the appearance of two new features (labeled G and H_c_) below *E*_F_. The peak with lower binding energy (G) is located at 0.76 eV, a little further away from *E*_F_, and is slightly wider than K-doped BBL. Nonetheless, these subtle changes of the occupied states cannot account for the large optical absorption differences, suggesting significant changes to the unoccupied electronic structure or the transition dipole moment. In contrast, Li-doped BBL produces a larger difference in the UPS spectra compared with the case of K doping, as shown in Figure S5. The new peak G is much stronger and sharper and shifts by the same amount (0.16 eV) toward higher binding energy as the secondary electron cut-off (SECO). *H*_a_ peak becomes flat after the first doping step and extends toward low binding energy, resulting in the absence of the peak *H*_c_. In addition, *H*_b_ exhibits a wider, multi-peak overlap structure in the doped spectrum. Despite these slight differences, the generation of the polaronic states and the downshift of the vacuum level are consistent in all three doping cases.

### Core Level Analysis

**Figure 3:**
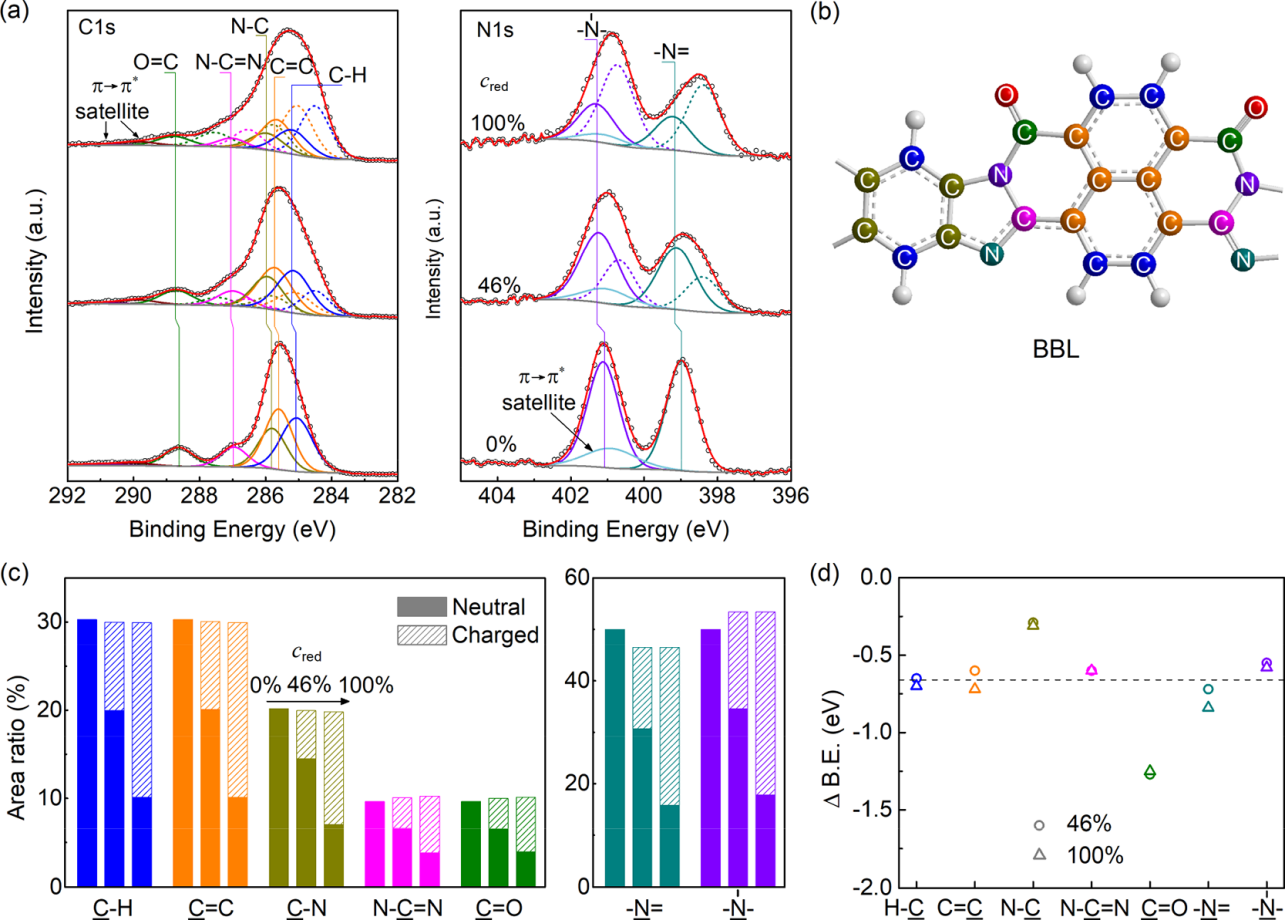
(a) High-resolution XPS C 1s (left) and N 1s (right) spectra of K-doped BBL at different doping ratios. (b) Unique color marks the corresponding atoms of each peak in the monomer. (c) Area ratio of all components is derived from the peak fitting. Three columns from left to right for each component corresponding to three doping ratios of 0, 46, and 100%. (d) Energy difference of each component between neutral and charged peaks.

To elucidate the chemical and electronic interaction between the polymer and K, we analyze the evolution of the core level spectra upon K doping. Figure 3a displays the C 1s and N 1s XPS spectra of 0 (undoped), 46, and 100% doped BBL with a careful fit according to the chemical environment and stoichiometry, which conforms well to the reported fit results (additional XPS spectra are shown in Figure S6).^37^ For clarity, each type of carbon and nitrogen is marked with a unique color, as shown in Figure 3b. The C 1s spectrum of the pristine BBL is dominated by a main peak and two weak peaks at higher binding energy. The main peak centered at 285.6 eV is composed of aromatic carbons on the naphthalene and benzene rings, which are subdivided into carbons connected with (C–H) and without (C=C) hydrogen and connected with nitrogen (C–N). The area ratio of these three peaks conforms well to the stoichiometry ratio of 6:6:4. The other two peaks correspond to N–C=N (lower binding energy) and C=O (higher binding energy) with a ratio of 2:2. All area ratios are summarized in the left panel of Figure 3c with the label of 0%, and the exact values are presented in Table S1.

Upon 46% K doping, corresponding to 0.46 K atoms per monomer, the major changes observed from the C 1s spectrum are that the C=O peak is significantly decreased in intensity, and the main peak is broadened toward low binding energy. This is in line with the fact that a portion of the polymer chains is n-doped by K. Accordingly, the spectrum is successfully fitted by the original five peaks from neutral BBL (solid lines in the middle-left panel of Figure 3a) and the other five peaks from the charged BBL (dashed lines). All neutral peaks are decreased by 33% and shifted by about 0.1 eV toward high binding energy, following the valence band shift with a similar amount. Each component of the charged C 1s compensates for the reduction of the corresponding neutral peak with a shift to the low binding energy, which is present in Figure 3c with the label of 46%. The perfect fit of the 100% K-doped spectrum using the same method but with an increased proportion of the charged BBL (66%) verifies the rationality of our analysis. The binding energy of each component is almost the same as the 46% doping ratio (see Table S1).

The N 1s spectrum (right panel of Figure 3a) of the pristine BBL shows two separate peaks, where the one at lower energy centered at 399.0 eV is assigned to the imine N and the other at higher energy centered at 401.1 eV is assigned to the amine N. The area ratio of the imine N is smaller than the stoichiometry ratio of 50% (2:2), in agreement with previous observations.^38^ We attribute it to the π–π* shakeup satellite of imine N that overlaps the amine N. For the 46% and 100% K-doping spectra, both peaks significantly broaden with their maxima shifting toward lower binding energies. These changes stem from the charged BBL that produces a new peak on the lower energy side of each original peak. The proportions of the new peaks are the same as those in C 1s, which are 33% at 46% K doping ratio and 66% at 100% K doping ratio, as shown in the right panel of Figure 3c. From these data, we find that the proportion of electron-charged monomers is less than the K doping ratio. This may be due to inhomogeneous doping, that is, some monomers are charged by more than one electron, or K aggregation, in which some K does not contribute to the doping.

Figure 3d shows the energy difference between the charged and neutral peaks of various types of carbon and nitrogen. We observe that the energy difference is similar between the two doping ratios but varies with the component. In detail, the binding energy of the C=O peak shows the most significant reduction of about 1.25 eV after being charged by an electron, indicating that significant extra electron density is located around the carbonyl group. The C–N peak shows the smallest binding energy difference, and the remaining components show a similar energy difference of about −0.7 eV. The same value of aromatic C 1s has been observed in Mg- and Li-doped PTCDA.^37,39^ This result also agrees with the DFT simulation that the polaronic state is mainly located on the benzophenanthroline unit, imine N, and O atoms. In contrast, the benzene ring’s four C atoms attached to N (C–N) contribute little to this state.^25^ From the simulation, we also observe that the amine has less contribution than the imine, resulting in the smaller energy difference of amino N (−0.60, −0.75 eV for the imine N).

A similar result is obtained from the XPS spectra of the pristine and K-doped BBB, as shown in Figure S7. The C 1s also consists of five components with an area ratio matching the stoichiometry. Note that the number of C atoms from C–H increases to 10 and from C=C increases to 8. However, the energy difference between the charged and neutral carbonyl C increases to about 1.5 eV, while the other four types of C show reduced shifts after being charged. This suggests that electrons are concentrated more on the carbonyl group than charged BBL chains, supporting the assumption that polarons are more localized in the ring-twisted BBB. We can infer that the vanishing of the neutral carbonyl C requires a doping ratio higher than 200% since there are two carbonyl groups in a repeat monomer as the feature is hard to be distinguished from the spectrum of 300% doping ratio, as shown in Figure S8. This is why the proportion of charged peaks is lower than that of BBL at the same doping ratio, which is also observed in the absorption and UPS measurement that BBB always uses a higher K content. By analyzing the core level, we find that K-doped BBL and BBB are typical n-type doping with the appearance of charged components in the low binding energy side. The only difference between the two cases is that the added charges are more localized in BBB.

### Electrical Conductivity Measurements

**Figure 4:**
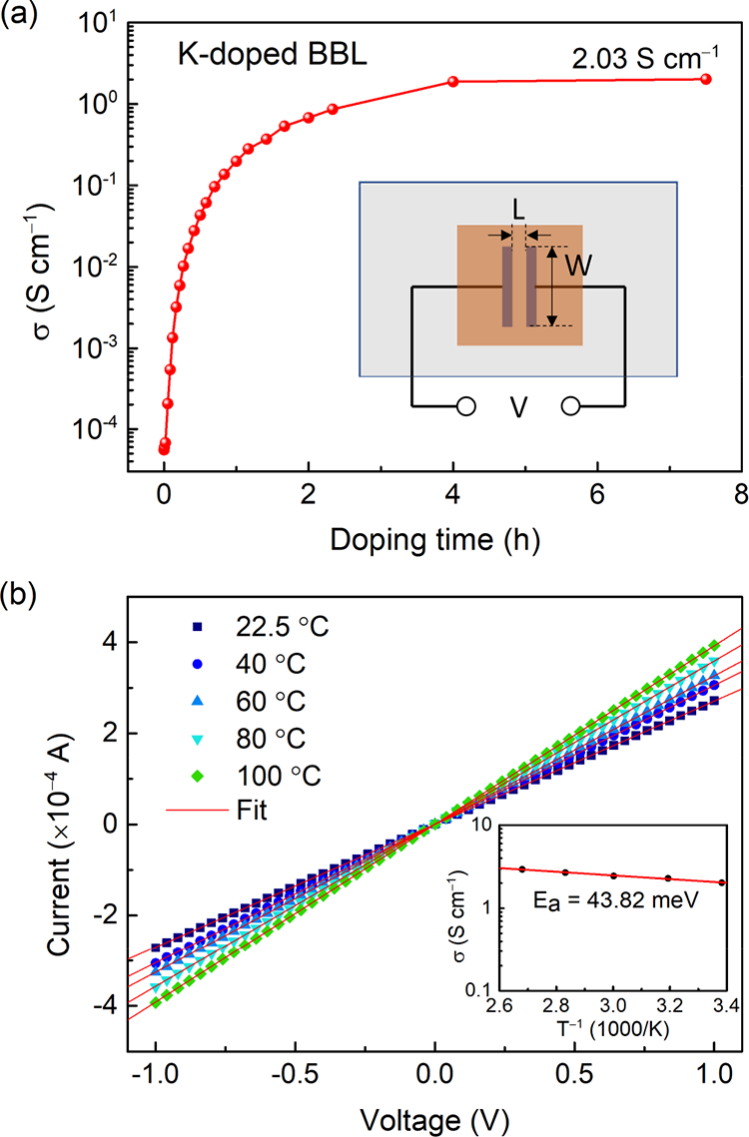
(a) Electrical conductivity of K-doped BBL as a function of the doping time. Inset shows the device structure for the electrical measurement. (b) Current–voltage curves of fully doped BBL at different temperatures. The electrical conductivities are summarized as a function of temperature (inset).

The formation of polarons in a conjugated polymer film usually facilities charge transport. Here, we investigate the electrical properties of alkali metal-doped BBL films by a two-probe method in UHV. The doping time dependence of the electrical conductivity is shown in Figure 4a. The conductivity of undoped BBL film is 5.5 × 10^–5^ S cm^–1^ and grows rapidly with increasing doping time. After 4 h doping, the electrical conductivity is stuck on a plateau of slow growth and reaches the maximum value of 2.03 S cm^–1^ at the final doping step (7.5 h). This value is slightly higher than the conductivity of molecularly doped BBL films (1.7 S cm^–1^ using TDAE as the dopant molecule), again showing the effective doping of K.^18,23^ By heating the doped film, we investigated the temperature dependence of the electrical conductivity, as shown in Figure 4b. The current–voltage curve at each temperature is collected after sufficient stabilization time. The activation energy derived from the Arrhenius plot (see the inset of Figure 4b) is 43.82 meV, an ultra-small value that has never been observed in the doped BBL film and is comparable to the n-doped fullerenes with disorder-free polaron transport.^40^ Based on the PES analysis and the GIWAXS data, which shows a strong (010) peak in the out-of-plane direction reported before,^23^ we attribute the small *E*_a_ to the highly ordered π–π stacking and the extended delocalization of the polaron.

The Li-doped BBL films, in contrast, show a much higher electrical conductivity of 37.09 S cm^–1^ after 3 h exposure to Li vapor (see Figure S9). This could be possibly due to the smaller size of Li^+^ cations that preserves the originally ordered nanostructure of BBL films to a greater extent, which requires further study for clarification. The electrical conductivity drops momentarily twice during the doping process, at 0.01 and 0.4 S cm^–1^. A similar result has been observed for electrochemically doped BBL^22^ and attributed to the multiple redox states. The same effect does not occur when using K or small organic molecules as dopants. Different from the excellent stability of K-doped BBL films in the UHV (Figure S10), the Li-doped BBL films are unstable. As shown in Figure S11, the electrical conductivity drops to 25.05 S cm^–1^ after 1 h in the UHV, and heating further accelerated the decline. This difference may be traced back to the interactions between the BBL chain and alkali metals.

### Dedoping by Air Exposure

**Figure 5:**
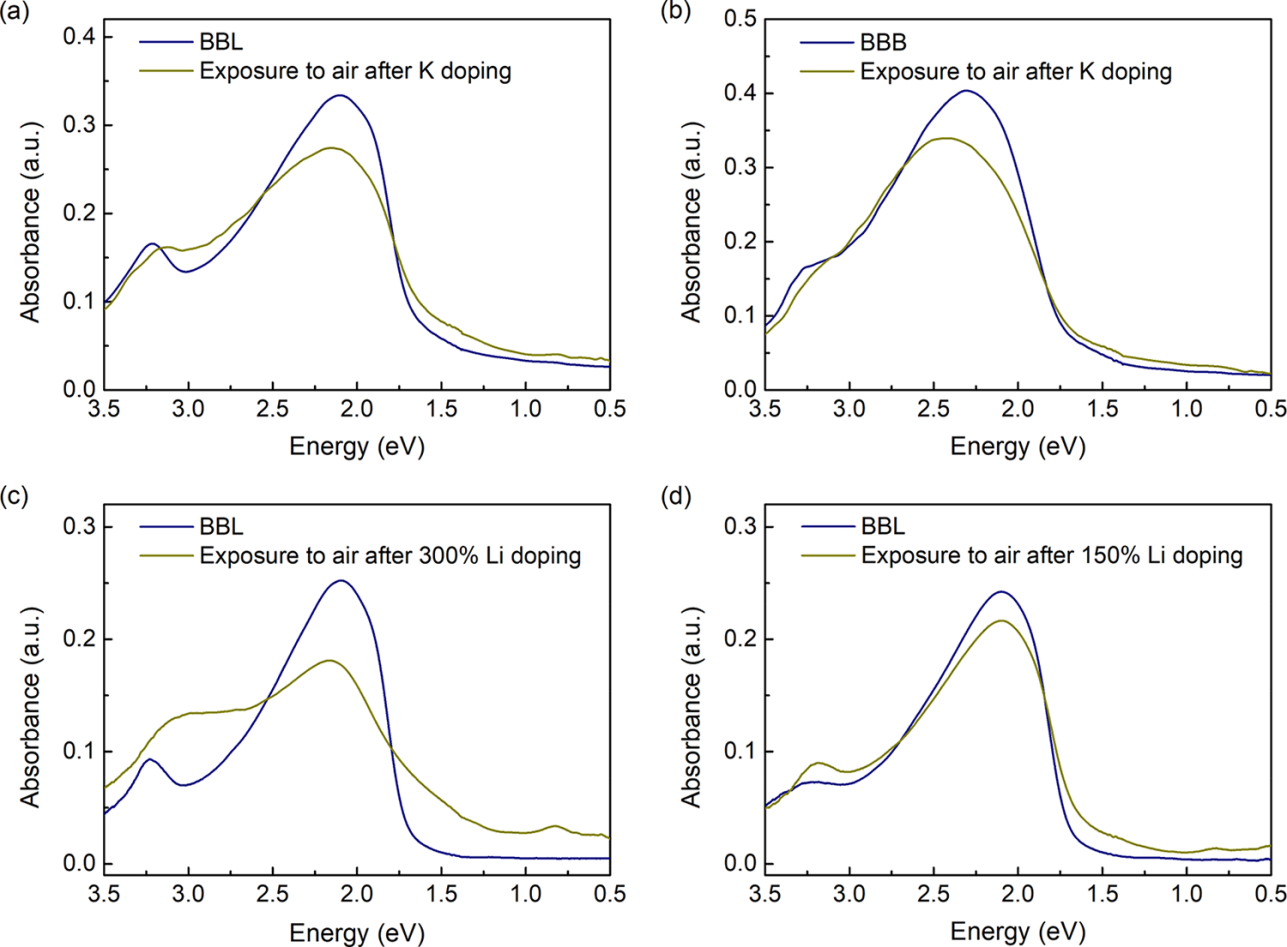
UV–vis–NIR spectra comparison between the pristine film and the doped film exposed to air for (a) K-doped BBL, (b) K-doped BBB, (c) 300% Li-doped BBL, and (d) 150% Li-doped BBL.

To further explore the interaction between the alkali metals and polymers, the doped films were exposed to air to remove the negative polarons by oxygen. After that, we repeated all the measurements in UHV by returning the films to the chamber. For UV–vis–NIR absorption, the spectra of K-doped films are mostly restored, as shown in Figure 5a,b, indicating that the reduction process is reversible within our doping ratios. Minor differences in the spectra, for example, the decrease in the neutral band A, may be caused by the residual polarons in the bulk protected by the upper layers of the film. For Li-doped BBL films, the spectrum is still essentially different from the pristine one after exposure to air (Figure 5c). Note that the doping ratio of the film is about 300%. We also measure the film with a 150% doping ratio, as shown in Figure 5d, lacking absorption in the NIR region. It recovers well after exposure to air, similar to the K-doped films.

For UPS measurements, the polaronic states thoroughly vanish from the spectra of all four doping cases after exposure to air, as shown in Figure S12. Besides, the K-doped BBL and K-doped BBB spectra are restored to those of the undoped films, while the Li-doped BBL (*c*_red_ = 300%) films show a completely changed spectrum. In contrast, the lower doped films (150% Li-doped BBL) show a much better-restored spectrum, as shown in Figure S12d. This phenomenon is also observed from the C 1s and N 1s core levels (see Figure S13) and consists well with the optical observations. These results suggest that Li features a different interaction with BBL compared to K. Due to the smaller size and higher ionization energy of Li compared to K, we speculate that Li^+^ cations may form covalent bonds during the formation of polarons at higher doping ratios. It is somewhat similar to the electrochemical process of PTCDA in the Li-ion battery, which is reversible for the reduction of the first two carbonyls and irreversible for higher reduction levels,^41^ but different from the electrochemical behavior of BBL in the Li ion battery.^42^ In the electrical measurement, the conductivity of K-doped BBL drops immediately to 0.15 S cm^–1^ after exposure to air and further decreases as the exposure time increases, as shown in Figure S10. Nonetheless, it is still 3 orders of magnitude higher than that of the pristine films and attributed to unoxidized polarons near the bottom-contact electrode of the device, which is consistent with the residual absorption of the bulk-doped BBL.

## Conclusions

We have explored the charged states resulting from n-doping of the prototypical ladder-type conjugated polymer BBL via systematic in situ spectroscopic and electrical characterizations. Using strong n-type dopants K and Li, we successfully detected the polaronic states above the Fermi level and new absorption bands across the entire UV–vis–NIR regions. The UV–vis–NIR and UPS spectra evolutions upon sequential alkali-metal doping provide a clear diagram for the frontier orbitals of multiply charged BBL. The interaction between the BBL chains and K is determined through XPS analysis. The electron distribution derived from the shift of each component after being charged agrees with the reported DFT calculations. Compared with the semiladder ring-twisted analogue BBB, a larger (electron) polaron extension is observed on the more planar BBL chains, resulting in the red-shift of the NIR absorption band and less K required to reach a similar reduction level. From the different performances after exposure to air, we find that the formed polarons in Li-doped BBL show more localized character than those in K-doped BBL, attributed to a more covalent bond formed for Li-doped BBL. Moreover, the electrical conductivity of K-doped BBL film shows the same trend as BBL doped by small organic molecules with a maximum value of 2.03 S cm^–1^. In contrast, Li doping shows two “dips” during the doping process and a higher final conductivity (37.09 S cm^–1^), which have only previously been observed in electrochemical reduction of BBL. These findings highlight the importance of polymer chain planarity and dopant size for the polaronic state in conducting polymers.

## Supporting Information Available

Schematic of the UHV system; molecular structures of *cis*- and *trans*-BBL; difference UV–vis–NIR and XPS spectra of K-doped BBL and BBB; UV–vis–NIR and UPS spectra of Li-doped BBL; electrical conductivity of Li-doped BBL; electrical stability of doped BBL; UPS and XPS comparison between pristine films and doped films exposed to air; and C 1s peak fitting parameters of the K-doped BBL (PDF)
