# Supplementary material for: In Situ Spectroscopic and Electrical Investigations of Ladder-type Conjugated Polymers Doped with Alkali Metals
Source: Macromolecules. 2022 Aug 15;55(16):7294–302. doi: 10.1021/acs.macromol.2c01190 (PMC9407040; doi:10.1021/acs.macromol.2c01190)
Supplement: Supplementary file 1 — ma2c01190_si_001.pdf [file ma2c01190_si_001.pdf]

Supporting information

In-situ Spectroscopic and Electrical Investigations of Ladder-Type Conjugated Polymers Doped with Alkali Metals

Yongzhen Chen,[1] Han-Yan Wu,[1] Chi-Yuan Yang,[1] Nagesh B. Kolhe,[2] Samson A. Jenekhe,[2] Xianjie Liu,[1] Slawomir Braun,[1] Simone Fabiano[1] and Mats Fahlman[1]*

[1] Laboratory of Organic Electronics, Department of Science and Technology, Linköping University, 60174 Norrköping, Sweden.

[2] Department of Chemical Engineering and Department of Chemistry, University of Washington, Seattle, WA 98195-1750, USA.

*Corresponding author: cyzljt@hotmail.com

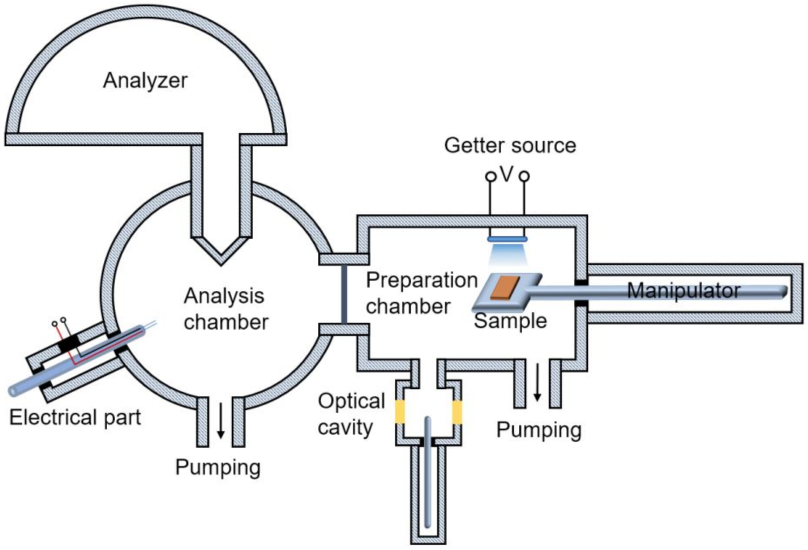

Figure S1. Schematic diagram of the in-situ doping and characterization system.

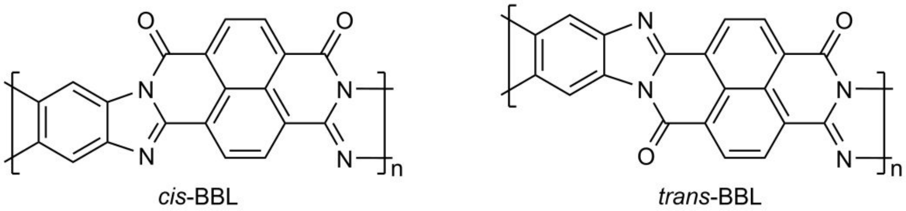

Figure S2. The structures of two conformers: *cis*-BBL (left) and *trans*-BBL (right).

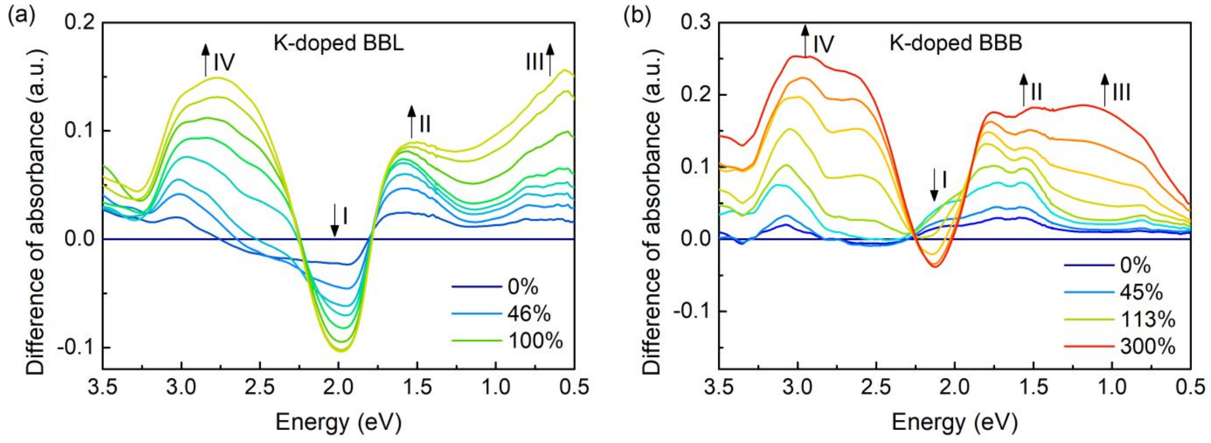

Figure S3. Difference UV−vis−NIR spectra of K-doped (a) BBL and (b) BBB films with the increase of doping ratio. The spectrum at each doping ratio is obtained by subtracting the pristine (0%) spectrum from the corresponding measured spectrum in Figure 1.

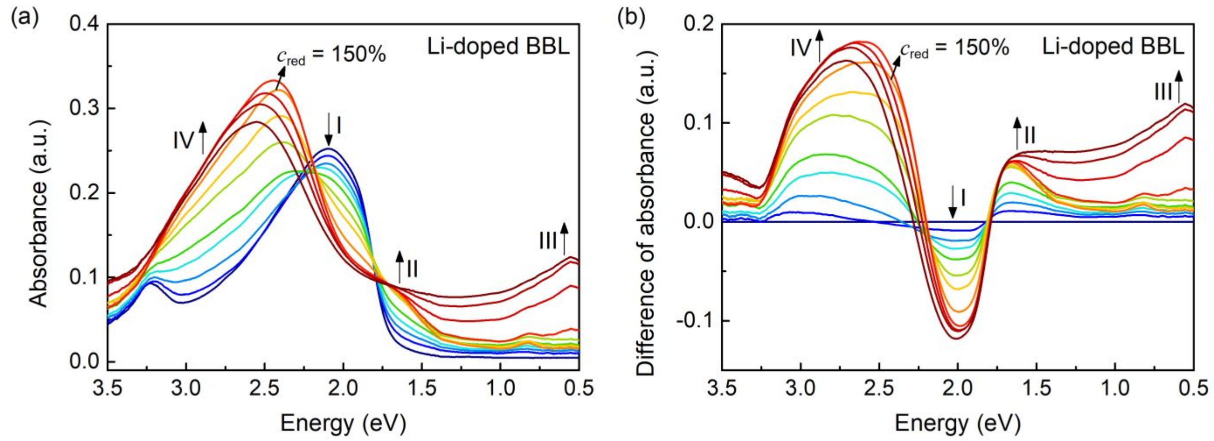

Figure S4. (a) UV−vis−NIR spectra and (b) corresponding difference spectra of the Li-doped BBL film with the increase of doping ratio. Only one doping ratio is marked in the figure.

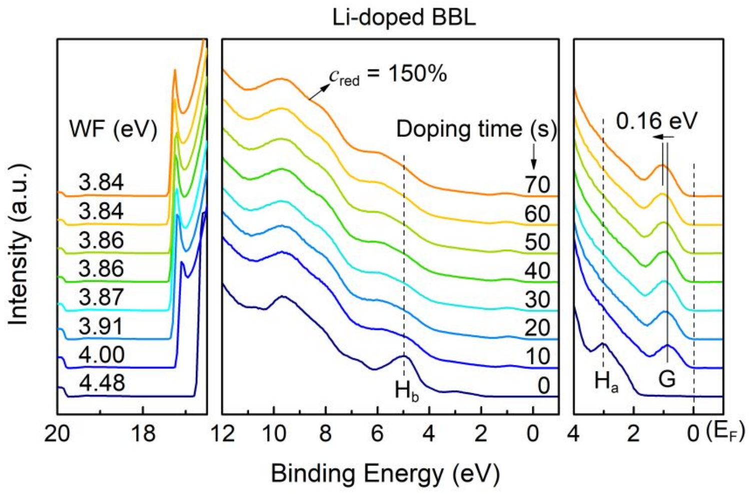

Figure S5. Evolution of UPS spectra of BBL with the increase of Li doping ratio. Due to the weak intensity of Li 1s, only the final doping ratio is obtained from XPS. The doping time is marked as a reference.

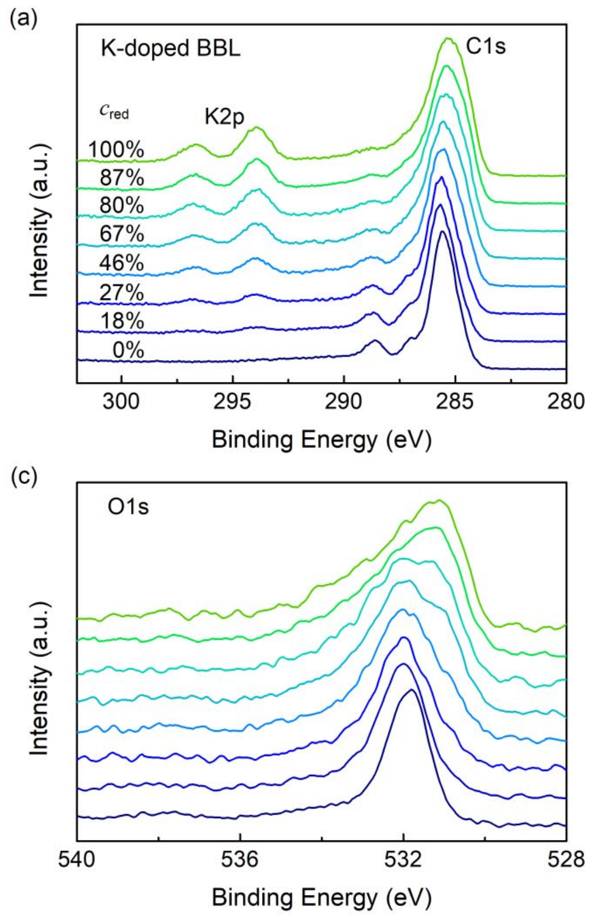

Figure S6. Evolution of XPS (a) C 1s, (b) N 1s, (c) O 1s, (d) wide scan spectra of K-doped BBL with incremental increasing doping ratio. The doping ratios marked upon the spectra are derived from the area ratios (A) between the K 2p and C 1s, taking the number of C atoms per repeat monomer (N = 20 for BBL and N = 26 for BBB) and the sensitivity factors (I) into consideration, which can be expressed as: $c_{red} = \frac{A_K I_C}{A_C I_K} N$.

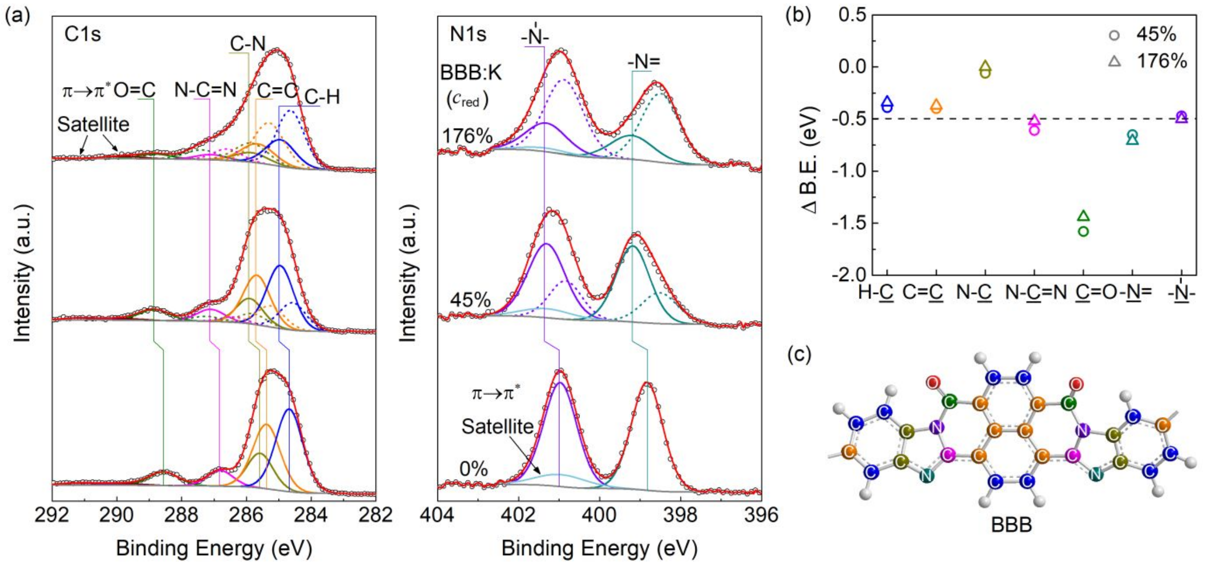

Figure S7. (a) High-resolution XPS C 1s (left) and N 1s (right) spectra of K-doped BBB at different doping ratios. (b) The energy difference of each component between neutral and charged peaks. (c) The corresponding atoms of each peak are marked by a unique color in the monomer.

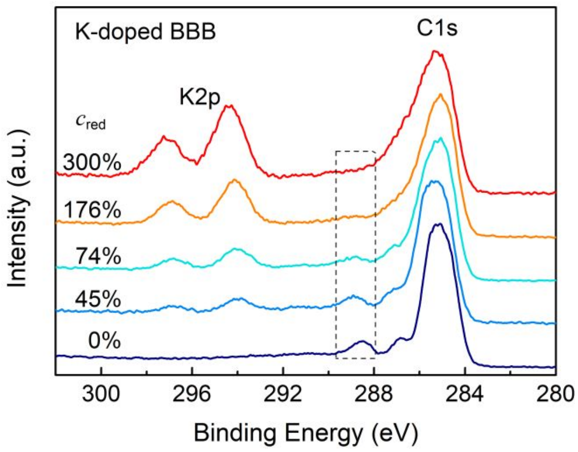

Figure S8. Evolution of XPS C 1s spectra of K-doped BBB with incremental increasing doping ratio.

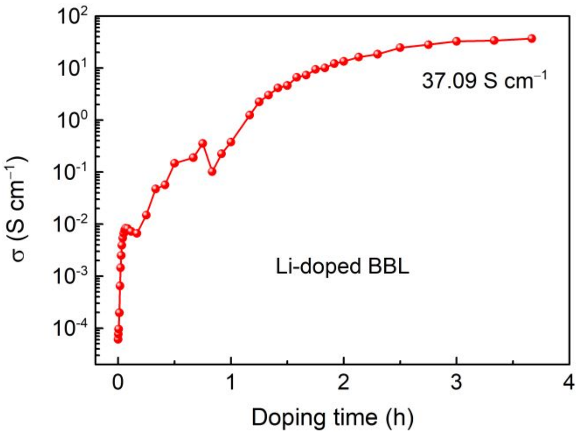

Figure S9. Electrical conductivity of BBL as a function of the Li doping time.

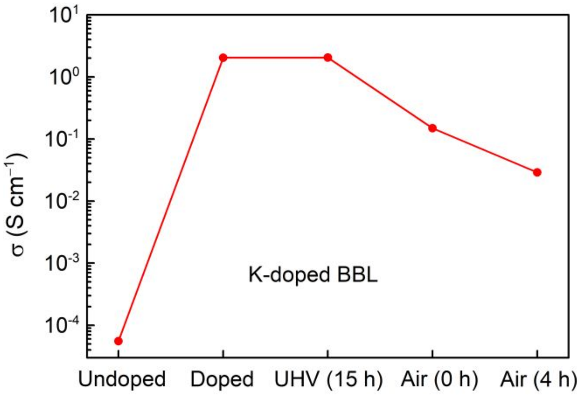

Figure S10. Electrical conductivities of undoped, K-doped BBL and the film staying 15 h in UHV, exposed to air after doping and staying 4 h in air, respectively.

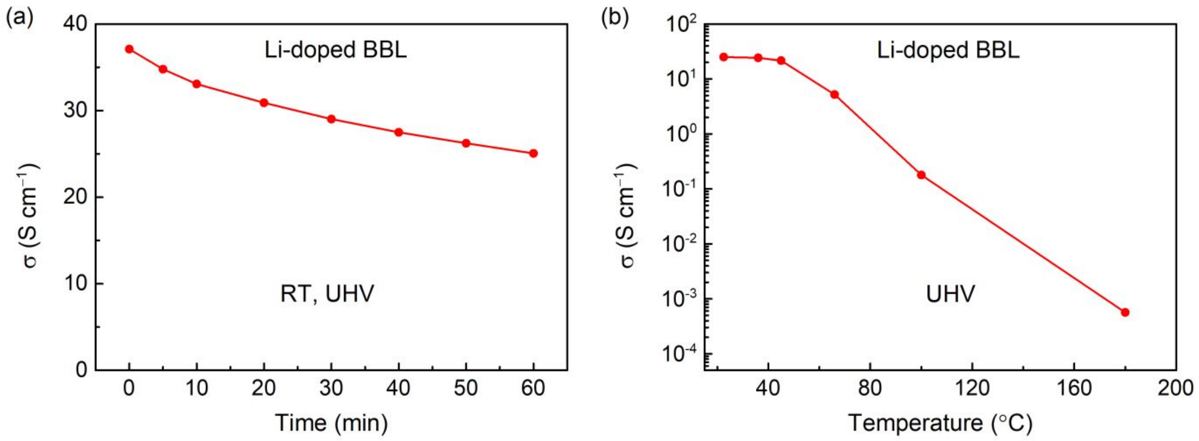

Figure S11. Electrical conductivity of Li-doped BBL changing with (a) staying time in UHV and (b) heating temperature.

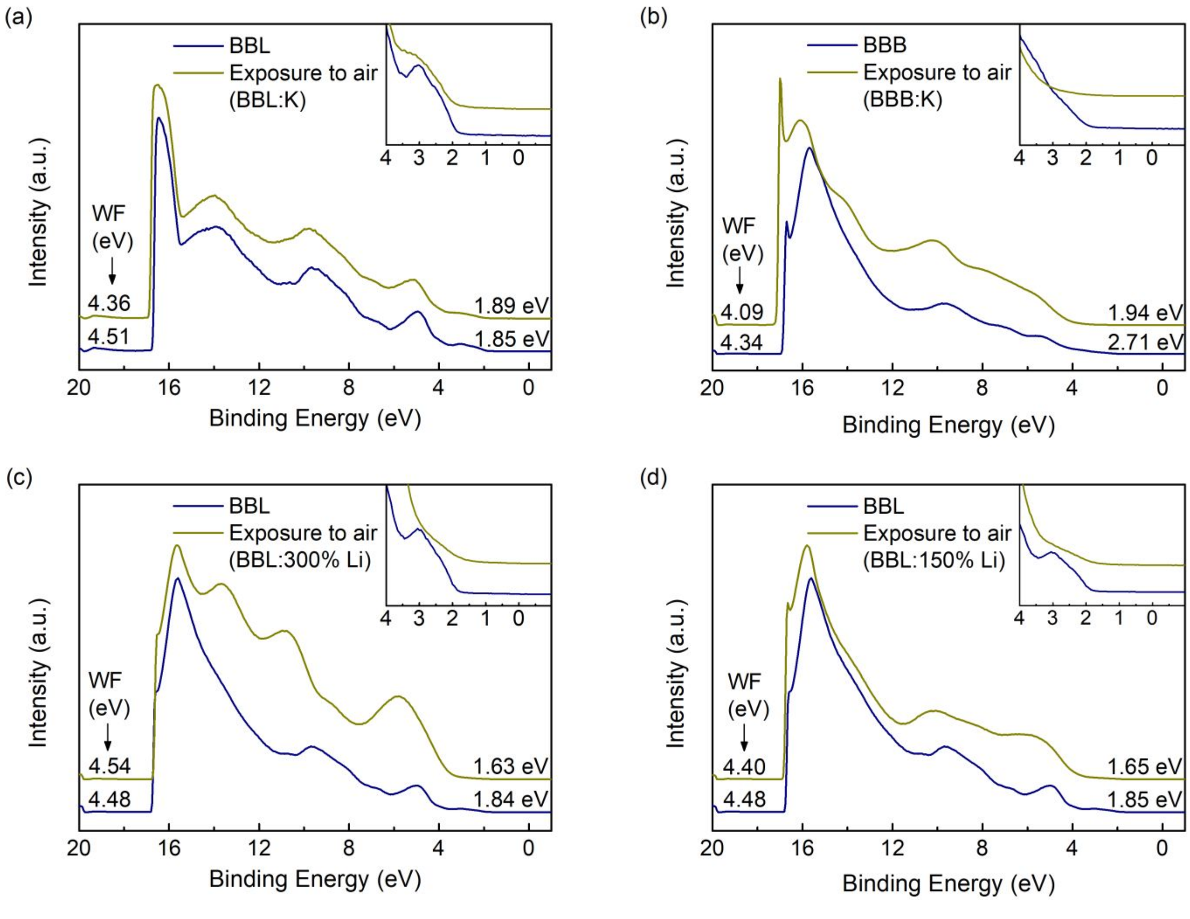

Figure S12. UPS comparison between the pristine film and doped film exposed to air for (a) BBL:K, (b) BBB:K, (c) BBL:300% Li and (d) BBL:150% Li. Insets show the enlarged valence band.

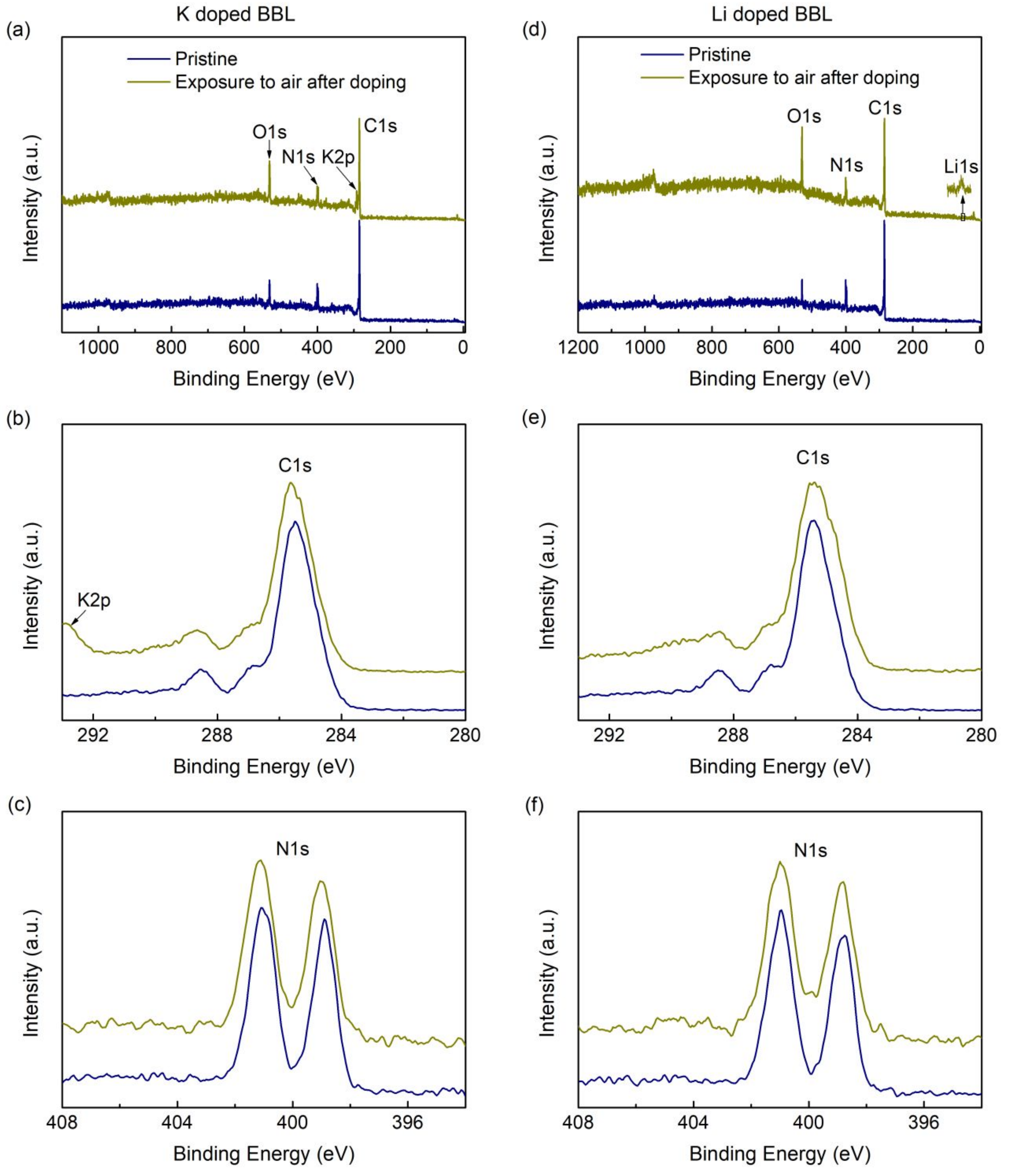

Figure S13. Core level comparison between the pristine film and doped film exposed to air for (a–c) K-doped BBL and (d–f) Li-doped BBL (c_{red} = 150%).

Table S1. C 1s peak fitting parameters of the K-doped BBL film including binding energy (B.E.), full width at half maximum (FWHM), area ratio and stoichiometric ratio.

Doping ratio	States	B.E. (eV)	FWHM (eV)	Area Ratio (%)	Stoichiometric Ratio (%)
0%	C–H	285.06	1.13	30.28	30
	C=C	285.60	1.00	30.28	30
	C–N	285.80	1.00	20.16	20
	N–C=N	286.96	0.97	9.64	10
	C=O	288.63	1.03	9.64	10
46%	C–H	285.15	1.20	19.98	30
		284.50	1.03	9.99	
	C=C	285.72	1.20	20.08	30
		285.12	1.22	9.99	
	C–N	285.94	1.08	14.49	20
		285.65	1.12	5.46	
	N–C=N	287.02	1.03	6.60	10
		286.42	1.05	3.47	
	C=O	288.71	1.15	6.52	10
		287.44	1.10	3.48	
100%	C–H	285.20	1.15	10.12	30
		284.50	1.10	19.80	
	C=C	285.76	1.06	10.12	30
		285.04	1.10	19.80	
	C–N	285.97	1.20	7.04	20
		285.66	1.10	12.76	
	N–C=N	287.06	1.00	3.87	10
		286.46	1.00	6.38	
	C=O	288.76	1.15	3.96	10
		287.51	1.22	6.16	
